# CircSERPINE2 weakens IL-1β-caused apoptosis and extracellular matrix degradation of chondrocytes by regulating miR-495/TGFBR2 axis

**DOI:** 10.1042/BSR20201601

**Published:** 2020-11-03

**Authors:** Qingpu Zhang, Xiaomiao Qiao, Wenwei Xia

**Affiliations:** Department of Orthopedic, Huiji District People’s Hospital, Zhengzhou, Henan, China

**Keywords:** osteoarthritis, IL-1β, chondrocytes, circSERPINE2, TGFBR2, miR-495

## Abstract

The dysregulated circular RNAs (circRNAs) are relevant to the development of osteoarthritis (OA). The circRNA serpin family E member 2 (circSERPINE2) is dysregulated in OA, while the role and mechanism of circSERPINE2 in OA are largely unknown. The aim of our research is to explore how and whether circSERPINE2 regulates interleukin-1β (IL-1β)-caused chondrocyte damage in OA. In the present study, the chondrocytes (CHON-001 cells) were exposed to IL-1β to mimic the injury in OA. CircSERPINE2, microRNA-495 (miR-495) and transforming growth factor-β receptor 2 (TGFBR2) abundances were detected via quantitative reverse-transcription polymerase chain reaction (qRT-PCR) or Western blot. Cell apoptosis was assessed via viability, apoptotic rate and caspase-3 activity. Extracellular matrix was investigated by levels of Sry-type high-mobility-group box 9 (SOX9), collagen type II α 1 (COL2A1) and Aggrecan using Western blot. The interaction among circSERPINE2, miR-495 and TGFBR2 was assessed via dual-luciferase reporter analysis and RNA immunoprecipitation (RIP). The results showed that circSERPINE2 expression was reduced in OA patients and IL-1β-treated chondrocytes. CircSERPINE2 overexpression mitigated IL-1β-caused apoptosis and extracellular matrix degradation. miR-495 was targeted by circSERPINE2 and up-regulated in OA patients and IL-1β-treated chondrocytes. miR-495 up-regulation reversed overexpression of circSERPINE2-mediated inhibition of apoptosis and extracellular matrix degradation. TGFBR2 was targeted by miR-495 and lowly expressed in OA patients and IL-1β-treated chondrocytes. CircSERPINE2 could mediate TGFBR2 expression by binding with miR-495. As a conclusion, circSERPINE2 attenuated IL-1β-caused apoptosis and extracellular matrix degradation of chondrocytes by regulating miR-495/TGFBR2 axis, indicating a new target for OA treatment.

## Introduction

Human osteoarthritis (OA) is characterized via chronic synovial inflammation, destruction of articular cartilage, and bone remodeling [1]. The chondrocytes apoptosis and extracellular matrix degradation are relevant to the destruction of articular cartilage, contributing to the progression of OA [2–4]. However, there is a poor understanding of how chondrocytes apoptosis and extracellular matrix degradation are triggered. Interleukin-1β (IL-1β) is required and involved in the injury of various cell types including chondrocytes in OA [5]. Thus, exploring the mechanism underlying IL-1β-induced chondrocytes injury might help provide new insight into the pathology of OA.

The noncoding RNAs have important roles in cartilage development and chondrocyte function [6,7]. Circular RNAs (circRNAs) are a type of RNAs characterized via covalently closed loop structure without 5′ caps and 3′ poly(A) tails, which take part in the development of OA [8]. For instance, hsa_circ_0005105 could facilitate extracellular matrix degradation of chondrocytes by up-regulating nicotinamide phosphoribosyl transferase (NAMPT) via sponging miR-26a [9]. Moreover, hsa_circ_0045714 could promote proliferation and extracellular matrix synthesis of chondrocytes by increasing miR-193b-targeted insulin-like growth factor 1 receptor [10]. In an emerging report, the investigators measured 25 most differentially expressed circRNAs, and confirmed that the circRNA serpin family E member 2 (circSERPINE2) derived from *SERPINE2* gene is a protective circRNA in OA by decreasing chondrocytes apoptosis and extracellular matrix degradation through miR-1271/ETS-related gene axis [11]. Although circSERPINE2 could participate in the regulation of OA progression, the mechanisms are complex. Thus, more regulatory networks addressed via circSERPINE2 in chondrocyte injury are needed to be explored.

microRNAs (miRNAs) are ∼22-nucleotide noncoding RNAs interacting with mRNAs by binding with their 3′UTR, which are involved in the development of OA [12]. MicroRNA-495 (miR-495) is a noncoding RNA located on chromosome 14, which is implicated in various processes in human diseases [13]. A previous study suggests that miR-495 contributes to chondrocytes apoptosis and OA progression via decreasing protein kinase Bα (AKT1) [14]. Nevertheless, whether circSERPINE2 can interact with miR-495 is still uncertain. Transforming growth factor-β receptor 2 (TGFBR2) is associated with the alteration of chondrocyte phenotype in OA [15]. Furthermore, increasing evidences report that TGFBR2 can inhibit OA-like phenotype in mice and IL-1β-challenged chondrocytes [16–18]. Bioinformatics analysis predicts both circSERPINE2 and TGFBR2 can bind with miR-495. Therefore, we hypothesize circSERPINE2 may regulate miR-495/TGFBR2 axis in chondrocytes.

In this research, we established IL-1β-challenged chondrocytes model, and investigated the function of circSERPINE2 on IL-1β-caused chondrocytes injury by detecting cell apoptosis and extracellular matrix degradation. Furthermore, we explored the interaction between circSERPINE2 and miR-495/TGFBR2 axis.

## Materials and methods

### Patients and tissue collection

Thirty-five OA patients and ten normal control patients without symptoms of OA who underwent the accidental injury were recruited from Huiji District People’s Hospital. The OA was diagnosed following the criteria of American College of Rheumatology [19]. The cartilage tissues were collected by the same team of orthopedists with a large amount of experience during knee replacement surgery, and stored at −80°C. All patients provided the written informed consent. This experiment was approved by the Ethics Committee of Huiji District People’s Hospital.

### Cell culture and treatment

Human chondrocytes (CHON-001 cells) were provided via American Type Culture Collection (Manassas, VA, U.S.A.) and grown in DMEM (Thermo Fisher, Waltham, MA, U.S.A.) plus 10% fetal bovine serum (HyClone, Logan, UT, U.S.A.) and 0.1 mg/ml G-418 (Thermo Fisher) in 5% CO_2_ at 37°C.

To mimic OA-like chondrocytes injury, CHON-001 cells were challenged via 10 ng/ml of IL-1β (Sangon Biotech, Shanghai, China) for 24 h as previously reported [20]. The cells in control group were treated with equal volumes of PBS.

### Cell transfection

CircSERPINE2 overexpression vector was synthesized via cloning circSERPINE2 (hsa_circ_0008365) sequence into pcDNA3.1 circRNA mini vector, with the pcDNA3.1 circRNA mini vector (Addgene, Cambridge, MA, U.S.A.) as negative control (pcDNA). siRNA for circSERPINE2 (si-circSERPINE2, 5′-UCCGGUGUGGUCGUCCUUGGU-3′), negative control of siRNA (si-con, 5′-AAGACAUUGUGUGUCCGCCTT-3′), miR-495 mimic (5′-AAACAAACAUGGUGCACUUCUU-3′), negative control of mimic (miR-con, 5′-ACGUGACACGUUCGGAGAATT-3′), miR-495 inhibitor (anti-miR-495, 5′-AAGAAGUGCACCAUGUUUGUUU-3′), and negative control of inhibitors (anti-miR-con, 5′-CAGUACUUUUGUGUAGUACAA-3′) were synthesized via Ribobio (Guangzhou, China). For cell transfection, CHON-001 cells with 60% confluence were transfected with the vectors or oligonucleotides (30 nM) using Lipofectamine 2000 (Thermo Fisher) for 24 h.

### Quantitative reverse-transcription polymerase chain reaction

The cartilage samples or CHON-001 cells were lysed in TRIzol solution (Solarbio, Beijing, China), and then RNA was extracted according to the acidguanidinium thiocyanate–phenol–chloroform extraction method [21]. The RNA in nuclear or cytoplasm was isolated using a Cytoplasmic & Nuclear RNA Purification kit (Norgen Biotek, Thorold, Canada). The RNA was reverse-transcribed to cDNA using specific reverse transcription kit (Fulengen, Guangzhou, China). The generated cDNA was diluted and applied to quantitative reverse-transcription polymerase chain reaction (qRT-PCR) via mixing with SYBR (Solarbio) and specific primers (Genscript, Nanjing, China). The primers were shown as: circSERPINE2 (hsa_circ_0008365) (sense, 5′-CGGGAAATCCTATCAAGTGC-3′; antisense, 5′-ATTGAAGTGGGAGCAGATGG-3′), SERPINE2 (sense, 5′-CCTCTTTCCGGCTGTGACC-3′; antisense, 5′-CCGTGGTAGGGCAGTTCAAT-3′), TGFBR2 (sense, 5′-GTTGGCGAGGAGTTTCCTGTT-3′; antisense, 5′-GTCCTATTACAGCTGGGGCA-3′), miR-495 (sense, 5′-TCCGATTCTTCACGTGGTAC-3′; antisense, 5′-GTGCAGGGTCCGAGGT-3′), U6 (sense, 5′-CTCGCTTCGGCAGCACA-3′; antisense, AACGCTTCACGAATTTGCGT), and GAPDH (sense, 5′-GAAAGCCTGCCGGTGACTAA-3′; antisense, 5′-TTCCCGTTCTCAGCCTTGAC-3′). U6 (for miR-495 or nuclear) or GAPDH (for circSERPINE2, SERPINE2, TGFBR2 or cytoplasm) served as a reference control. Relative RNA level was calculated using 2^−ΔΔ*C*_t_^ method [22].

### The validation of circRNA circularization

RNase R could digest linear RNAs, but shows little effect on circRNAs. The isolated RNA was incubated with RNase R (4 U/μg; Geneseed, Guangzhou, China) for 30 min at 37°C. Next, the levels of circSERPINE2 and linear SERPINE2 were examined via qRT-PCR.

The random primers are suitable for the reverse transcription of almost all RNA, but Oligo(dT)18 primers are applied to the reverse transcription of RNA with poly(A) tail. CircRNAs have no poly(A) tails. During the reverse transcription, the random or Oligo(dT)18 primers were used, respectively.

### Cell viability, caspase-3 activity and flow cytometry

Cell viability was detected via 3-(4,5-dimethyl-2-thiazolyl)-2,5-diphenyl-2–H -tetrazolium bromide (MTT) analysis. A total of 1 × 10^4^ CHON-001 cells were placed into 96-well plates in quadruplicate overnight and then stimulated via 10 ng/ml of IL-1β for 24 h. Next, the medium was discarded and changed to fresh one plus 0.5 mg/ml MTT (Beyotime, Shanghai, China). After culture for 4 h, the medium was replaced with 100 μl of DMSO (Beyotime). The absorbance at 570 nm was examined using a microplate reader (Molecular Devices, Sunnyvale, CA, U.S.A.). Cell viability was normalized to the control group, and the control group was regarded as 100%.

For detection of caspase-3 activity, 4 × 10^5^ CHON-001 cells were placed into six-well plates in quadruplicate and then exposed to 10 ng/ml of IL-1β for 24 h. Next, cells were collected and lysed, and the cell lysates were used for caspase-3 activity assay via a caspase-3 assay kit (Abcam, Cambridge, MA, U.S.A.) according to the instructions of manufacturer. The optical density value at 400 nm was measured using a microplate reader. The relative caspase-3 activity was normalized to the control group.

Cell apoptotic rate was tested using an Annexin V-FITC apoptosis detection kit (Thermo Fisher) via flow cytometry. A total of 2 × 10^5^ CHON-001 cells were added into six-well plates and then incubated with 10 ng/ml of IL-1β for 24 h. Each sample was prepared in quadruplicate. Next, cells were collected, interacted with binding buffer and then dyed with Annexin V-FITC and propidium iodide (PI), followed via the detection of cell apoptosis using a flow cytometer (Agilent, Hangzhou, China). The apoptotic rate of CHON-001 cells was expressed as the percentage of cells with Annexin V-FITC^+^ and PI^+/−^.

### Western blot

After lysing in RIPA buffer (Solarbio), cell lysates were centrifuged for protein collection. The concentration of protein sample was tested using a BCA kit (Abcam). Twenty microgram samples were loaded on sodium dodecyl sulfate/polyacrylamide gel electrophoresis and then transferred to nitrocellulose membranes (Solarbio). Next, the membranes were blocked in 5% non-fat milk, and then interacted with primary antibodies anti-Sry-type high-mobility-group box 9 (SOX9) (ab3697, 1:1000 dilution, Abcam), anti-collagen type II α 1 (COL2A1) (AB761, 1:100 dilution, Sigma–Aldrich), anti-Aggrecan (AB1031, 1:1000 dilution, Sigma–Aldrich), anti-TGFBR2 (ab186838, 1:500 dilution, Abcam) or anti-β-actin (ab227387, 1:5000 dilution, Abcam) and the secondary antibody conjugated by horseradish peroxidase (ab205718, 1:20000 dilution, Abcam). β-actin functioned as a loading control. Subsequently, the protein blots were developed via exposing to ECL reagent (Solarbio) and tested by ImageJ software (NIH, Bethesda, MD, U.S.A.). Relative protein level was normalized to the control group.

### Dual-luciferase reporter analysis and RNA immunoprecipitation

The complementary sequence of circSERPINE2 and miR-495 was explored via CircInteractome (https://circinteractome.nia.nih.gov/), and that of miR-495 and TGFBR2 was searched via DIANA tools (http://diana.imis.athena-innovation.gr/DianaTools/index.php?r=microT_CDS/index). The luciferase reporter vectors circSERPINE2-WT or TGFBR2-WT were constructed via inserting the wildtype sequence of circSERPINE2 or TGFBR2 3′UTR into psiCHECK-2 vectors (YouBio, Changsha, China), respectively. The mutant-type luciferase reporter vectors circSERPINE2-MUT or TGFBR2-MUT were generated via using the corresponding mutant sequence. For dual-luciferase reporter analysis, CHON-001 cells were co-transfected with the constructed vectors or miR-495 mimic or miR-con for 24 h. Next, the luciferase activity was tested using a dual-luciferase analysis kit (Promega, Madison, WI, U.S.A.), and detected via a GloMax 20/20 Luminometer (Promega).

RNA immunoprecipitation (RIP) analysis was performed using a Magna RIP kit (Sigma, St. Louis, MO, U.S.A.). A total of 1 × 10^7^ CHON-001 cells were lysed, and the cell lysates were interacted with the magnetic beads conjugated via anti-protein argonaute-2 (Ago2) for 6 h. IgG was regarded as a negative control. The immunoprecipitated RNA on the beads was extracted, and the enrichment levels of circSERPINE2, miR-495 and TGFBR2 in the complex were examined via qRT-PCR.

### Statistical analysis

The data with normal distribution were shown as mean ± SD. The linear correlation among circSERPINE2, miR-495 and TGFBR2 expression in OA patients was tested via Pearson correlation analysis. The difference was compared via Student’s *t* test or ANOVA with Tukey’s test using GraphPad Prism 6 (GraphPad Inc., La Jolla, CA, U.S.A.). And it was statistically significant at *P*<0.05.

## Results

### CircSERPINE2 level is reduced in OA

**Figure 1:**
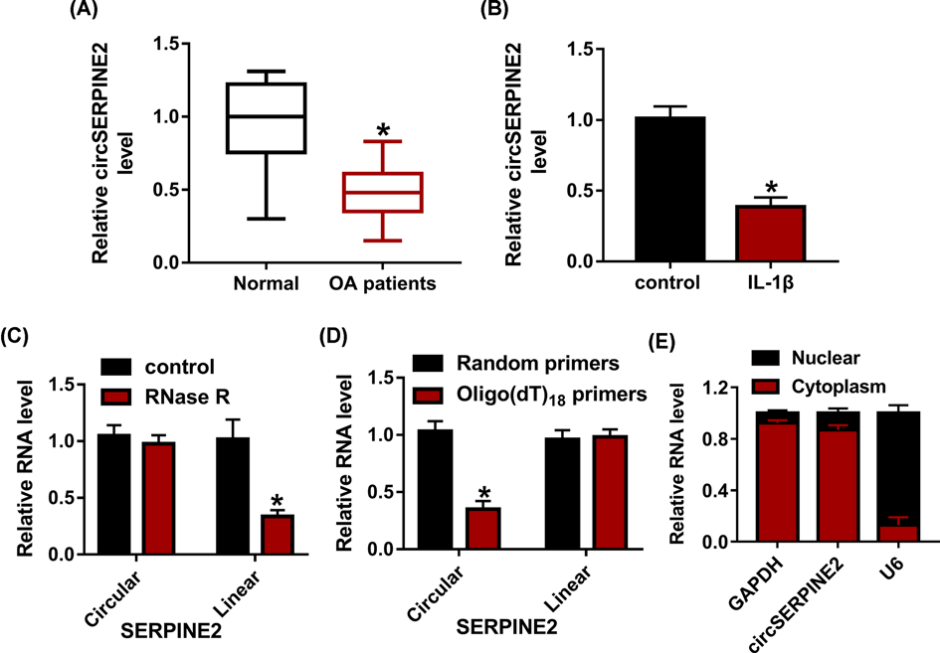
The expression of circSERPINE2 in OA (**A**) CircSERPINE2 expression was detected in cartilage tissues from OA patients (*n*=35) or normal patients (without OA, *n*=10). (**B**) CircSERPINE2 level was measured in IL-1β-challenged CHON-001 cells. (**C**) The circular and linear SERPINE2 were detected after treatment of RNase R. (**D**) The circular and linear SERPINE2 were measured with the reverse transcription using Random primers or Oligo(dT)18 primers. (**E**) CircSERPINE2 abundance was detected in nuclear and cytoplasm fractions. *P<0.05.

To measure the expression of circSERPINE2 in OA patients, the cartilage tissues from OA patients (*n*=35) or normal patients (without OA, *n*=10) were collected. CircSERPINE2 expression was evidently reduced in OA patients compared with normal subjects (Figure 1A). Moreover, IL-1β-challenged CHON-001 cellular model was established. CircSERPINE2 level was markedly declined in CHON-001 cells after exposure to IL-1β (Figure 1B). Additionally, the RNA from CHON-001 cells was treated with or without RNase R which could digest linear RNAs. Results showed that circSERPINE2 was more resistant to RNase R than linear SERPINE2 (Figure 1C). Furthermore, using random or Oligo(dT)18 primers followed via qRT-PCR, the circular SERPINE2 level was decreased using Oligo(dT)18 primers compared with random primers, suggesting that circSERPINE2 lacked the structure of poly(A) tail (Figure 1D). These confirmed the circularization of circSERPINE2. Besides, by separating the nuclear and cytoplasm, qRT-PCR assay showed that circSERPINE2 was mainly located in cytoplasm of CHON-001 cells (Figure 1E). These results indicated that circSERPINE2 might be involved in IL-1β-induced chondrocyte injury.

### Overexpression of circSERPINE2 alleviates IL-1β-induced apoptosis and extracellular matrix degradation

**Figure 2:**
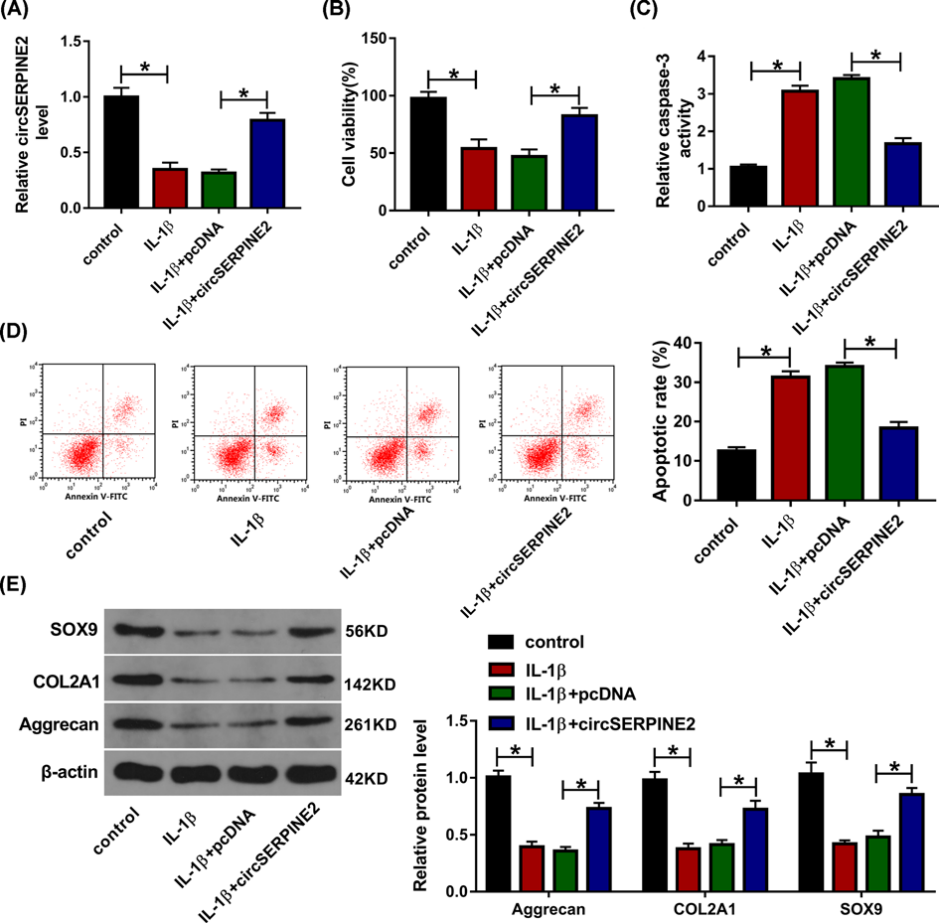
The effect of circSERPINE2 on IL-1β-induced chondrocyte injury CircSERPINE2 level (**A**), cell viability (**B**), caspase-3 activity (**C**), apoptotic rate (**D**), and protein levels of SOX9, COL2A1 and Aggrecan (**E**) were detected in CHON-001 cells transfected with pcDNA or circSERPINE2 overexpression vector after treatment of IL-1β. *P<0.05.

To explore the role of circSERPINE2 in IL-1β-induced chondrocyte injury, CHON-001 cells were transfected with pcDNA or circSERPINE2 overexpression vector and then exposed to IL-1β. As displayed in Figure 2A, circSERPINE2 level was evidently decreased in IL-1β-challenged CHON-001 cells, but it was up-regulated by transfection of circSERPINE2 overexpression vector. Moreover, MTT assay showed that the viability of CHON-001 cells was markedly reduced via exposure to IL-1β, which was restored via circSERPINE2 overexpression (Figure 2B). In addition, the caspase-3 activity and apoptotic rate in CHON-001 cells were evidently enhanced by treatment of IL-1β, and these events were weakened by circSERPINE2 overexpression (Figure 2C,D). Besides, the extracellular matrix-related markers were detected in IL-1β-challenged CHON-001 cells. Results displayed that the protein levels of SOX9, COL2A1 and Aggrecan were significantly reduced by IL-1β treatment, which was reversed via up-regulation of circSERPINE2 (Figure 2E). These data suggested that circSERPINE2 overexpression could mitigate IL-1β-induced chondrocyte injury.

### miR-495 is targeted via circSERPINE2

**Figure 3:**
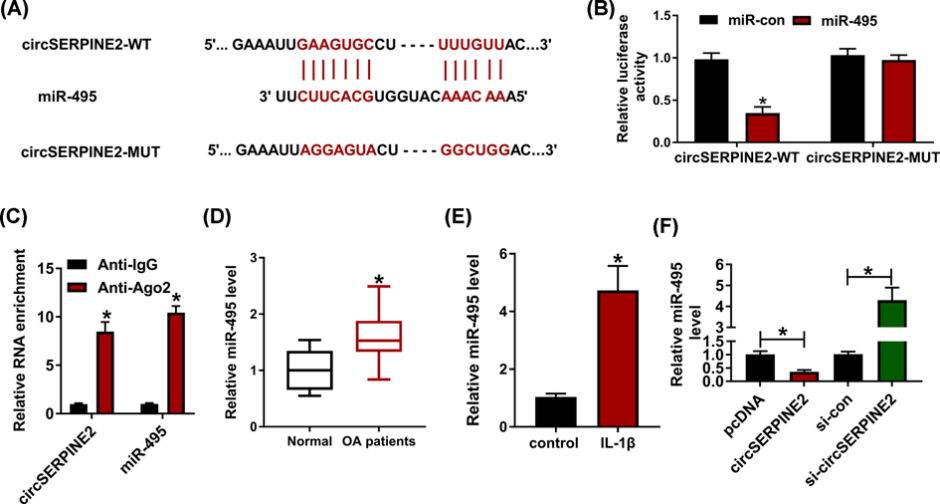
The relationship of circSERPINE2 and miR-495 (**A**) The binding sequence between circSERPINE2 and miR-495. (**B**) Luciferase activity was detected in CHON-001 cells co-transfected with circSERPINE2-WT or circSERPINE2-MUT and miR-495 mimic or miR-con. (**C**) CircSERPINE2 and miR-495 levels were detected after Ago2 or IgG RIP. (**D**) miR-495 expression was measured in OA patients and normal patients. (**E**) miR-495 level was detected in IL-1β-challenged CHON-001 cells. (**F**) miR-495 level was examined in CHON-001 cells transfected with pcDNA, circSERPINE2 overexpression vector, si-con or si-circSERPINE2. *P<0.05.

To explore the potential regulatory network mediated by circSERPINE2, the targeted miRNAs were searched by CircInteractome. miR-495, an OA-associated miRNA, was a predicted target of circSERPINE2, and their binding sequence was displayed in Figure 3A. To identify their correlation, the luciferase reporter vectors circSERPINE2-WT and circSERPINE2-MUT were conducted, and dual-luciferase reporter analysis was performed. The results showed that miR-495 overexpression caused more than 60% reduction in luciferase activity of circSERPINE2-WT, but it did not change the luciferase activity of circSERPINE2-MUT (Figure 3B). Furthermore, RIP assay was also used to validate the interaction between circSERPINE2 and miR-495. As shown in Figure 3C, there were amount of circSERPINE2 and miR-495 enriched in the same complex by Ago2 RIP. Additionally, miR-495 expression was detected in OA patients and IL-1β-treated cells. Results showed that miR-495 level was evidently increased in OA patients compared with normal group (Figure 3D). Similarly, miR-495 abundance in CHON-001 cells was elevated by 4.5-fold after exposure to IL-1β (Figure 3E). Besides, miR-495 level was markedly declined by circSERPINE2 overexpression and enhanced via circSERPINE2 knockdown (Figure 3F). These results indicated that circSERPINE2 could target miR-495 in chondrocytes.

### Up-regulation of miR-495 reverses the effect of circSERPINE2 on IL-1β-induced apoptosis and extracellular matrix degradation

**Figure 4:**
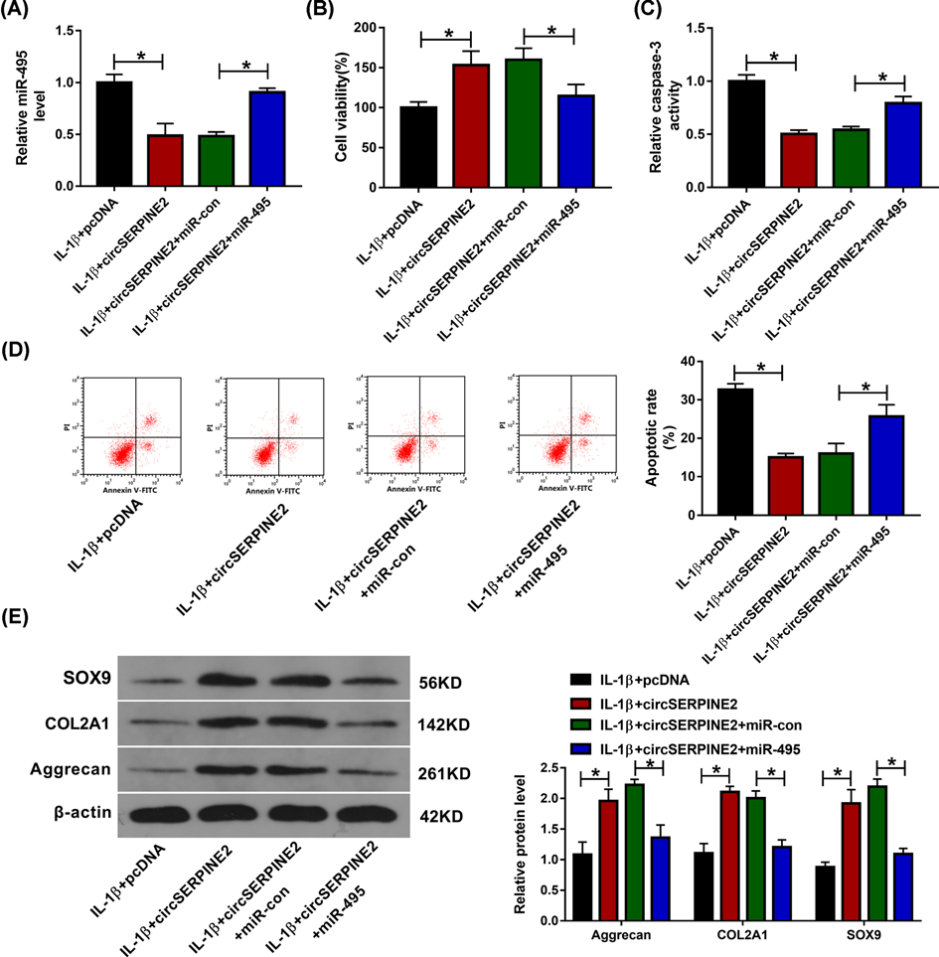
The effect of miR-495 on circSERPINE2-mediated regulation of IL-1β-induced chondrocyte injury miR-495 expression (**A**), cell viability (**B**), caspase-3 activity (**C**), apoptotic rate (**D**), and protein levels of SOX9, COL2A1 and Aggrecan (**E**) were examined in CHON-001 cells transfected with pcDNA, circSERPINE2 overexpression vector, circSERPINE2 overexpression vector + miR-con or miR-495 mimic after treatment of IL-1β. *P<0.05.

To explore whether circSERPINE2-mediated regulation of chondrocyte injury required miR-495, CHON-001 cells were transfected with pcDNA, circSERPINE2 overexpression vector, circSERPINE2 overexpression vector + miR-con or miR-495 mimic and then exposed to IL-1β. As exhibited in Figure 4A, miR-495 abundance was evidently reduced via circSERPINE2 overexpression in IL-1β-challenged CHON-001 cells, but it was restored via transfection of miR-495 mimic. Furthermore, up-regulation of miR-495 mitigated circSERPINE2-meditaed elevation of cell viability, and reduction in caspase-3 activity and apoptotic rate in IL-1β-challenged CHON-001 cells (Figure 4B–D). Additionally, restoration of miR-495 relieved the effect of circSERPINE2 on protein expression of SOX9, COL2A1 and Aggrecan in IL-1β-challenged cells (Figure 4E). These results indicated that circSERPINE2 could weaken IL-1β-induced chondrocyte injury by regulating miR-495.

### TGFBR2 is targeted via miR-495

**Figure 5:**
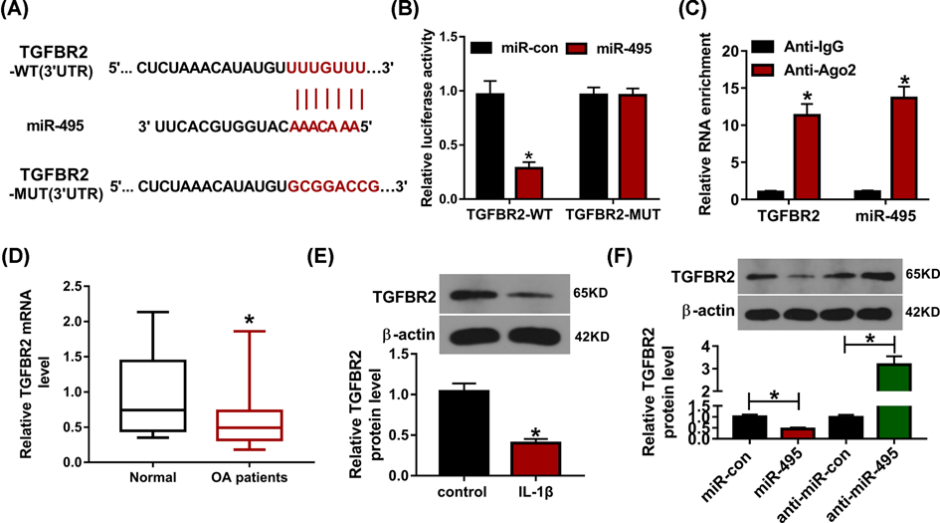
The relationship of miR-495 and TGFBR2 (**A**) The binding sequence between miR-495 and TGFBR2. (**B**) Luciferase activity was tested in CHON-001 cells co-transfected with TGFBR2-WT or TGFBR2-MUT and miR-495 mimic or miR-con. (**C**) miR-495 and TGFBR2 levels were measured after Ago2 or IgG RIP. (**D**) TGFBR2 mRNA expression was detected in OA patients and normal patients. (**E**) TGFBR2 protein level was measured in IL-1β-challenged CHON-001 cells. (**F**) TGFBR2 protein expression was examined in CHON-001 cells transfected with miR-con, miR-495 mimic, anti-miR-con or anti-miR-495. *P<0.05.

To further explore the circRNA/miRNA/mRNA network mediated via circSERPINE2/miR-495, the target of miR-495 was searched via DIANA tools. TGFBR2 was predicted as a candidate target of miR-495, and the binding sites of miR-495 and TGFBR2 were shown in Figure 5A. To validate this relationship, the luciferase reporter vectors TGFBR2-WT and TGFBR2-MUT were conducted, and transfected into CHON-001 cells. The data of dual-luciferase reporter assay displayed that miR-495 addition reduced ∼70% of luciferase activity of TGFBR2-WT, while it caused little effect on the luciferase activity of TGFBR2-MUT (Figure 5B). In addition, miR-495 and TGFBR2 were enriched in the same complex by Ago2 RIP (Figure 5C). Moreover, TGFBR2 expression was examined in OA patients and IL-1β-treated cells. As displayed in Figure 5D,E, TGFBR2 expression was evidently declined in OA patients compared with normal group and CHON-1 cells challenged via IL-1β. Besides, TGFBR2 protein abundance in CHON-001 cells was significantly decreased via miR-495 overexpression and elevated via miR-495 knockdown (Figure 5F). These findings suggested that miR-495 could target TGFBR2 in chondrocytes.

### CircSERPINE2 regulates TGFBR2 expression via miR-495

**Figure 6:**
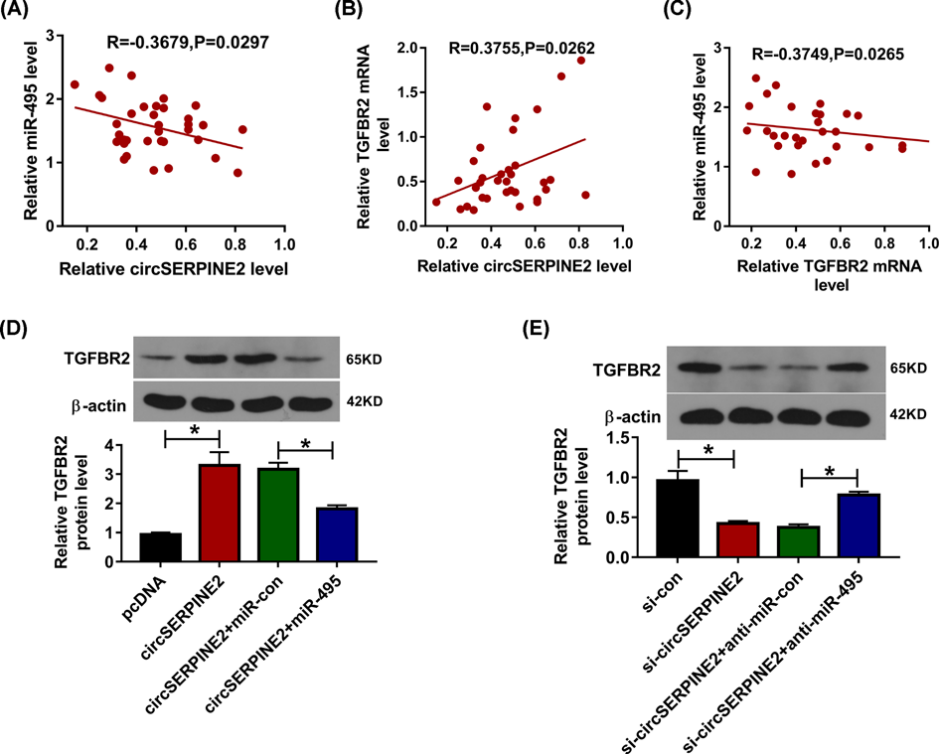
The relationship among circSERPINE2, miR-495 and TGFBR2 (**A–C**) The linear association among circSERPINE2, miR-495 and TGFBR2 levels in OA patients was analyzed. (**D,E**) TGFBR2 protein expression was measured in CHON-001 cells transfected with pcDNA, circSERPINE2 overexpression vector, circSERPINE2 overexpression vector + miR-con or miR-495 mimic, si-con, si-circSERPINE2, si-circSERPINE2 + anti-miR-con or anti-miR-495. *P<0.05.

To test whether circSERPINE2 could modulate TGFBR2 by miR-495, their correlations were analyzed. As displayed in Figure 6A,B, miR-495 level in OA patients were inversely correlated with circSERPINE2 and TGFBR2. Moreover, TGFBR2 expression in OA patients was positively associated with circSERPINE2 level (Figure 6C). In addition, the effect of circSERPINE2 and miR-495 on TGFBR2 expression was tested. The results showed that TGFBR2 protein expression was markedly enhanced by circSERPINE2 overexpression, which was weakened via miR-495 overexpression (Figure 6D). Besides, TGFBR2 protein abundance in CHON-001 cells was evidently declined via circSERPINE2 silence, which was restored via miR-495 knockdown (Figure 6E). These results indicated that circSERPINE2 could promote TGFBR2 abundance by regulating miR-495.

## Discussion

OA is one common type of arthritis in elderly populations, but the effective option for treatment of OA remains limited partly due to the incomplete understanding of OA pathogenesis [23]. The loss of extracellular matrix of articular cartilage is a key factor for OA progression [2]. Moreover, the chondrocytes apoptosis also occurs in OA development [3,4]. The dysregulated circRNAs are implicated in OA development and treatment [24]. In this research, we aimed to explore the function and mechanism of circSERPINE2 in chondrocytes apoptosis and extracellular matrix degradation. Our study confirmed that circSERPINE2 attenuated IL-1β-induced chondrocytes apoptosis and extracellular matrix degradation. Importantly, our study was the first to provide this was associated with the regulatory network of circSERPINE2/miR-495/TGFBR2.

We first measured circSERPINE2 expression in OA cartilage tissues and IL-1β-challenged chondrocytes, and found that circSERPINE2 abundance was reduced, which was also in agreement with a previous study [11]. This indicated the decreased circSERPINE2 might be associated with IL-1β-induced chondrocyte injury. Here we constructed IL-1β-induced OA cellular model. In IL-1β-challenged chondrocytes, the apoptosis and extracellular matrix degradation were induced, which was also similar as previously reported [25–27]. To explore the function of circSERPINE2, we performed the gain-of-function experiments by overexpressing circSERPINE2 in IL-1β-challenged chondrocytes. We found that circSERPINE2 overexpression mitigated IL-1β-induced chondrocytes apoptosis by decreasing caspase-3 activity and apoptotic rate, and alleviated IL-1β-induced extracellular matrix degradation by restoring the expression of COL2A1 and Aggrecan. This was also consistent with the report of Shen et al. [11]. Our results indicated that circSERPINE2 played a protective role in chondrocytes under IL-1β exposure, and suggested that circSERPINE2 might be used as a target for OA treatment.

The key mechanism addressed via circRNAs is to target mRNA by sponging miRNAs, and this network is involved in OA development [28]. Previous studies reported circSERPINE2 could exhibit its function via regulating miR-1271/ETS-related gene axis or miR-375/YWHAZ axis [11,29]. This study aimed to explore an additional regulatory network of circSERPINE2. Here we validated miR-495 was targeted via circSERPINE2. Yang et al. suggested that miR-495 knockdown could inhibit chondrocytes apoptosis by activating the NF-kB pathway in OA [30]. Moreover, Zhao et al. reported that miR-495 overexpression could promote chondrocytes apoptosis and extracellular matrix degradation via decreasing AKT1 [14]. These implied the promoting role of miR-495 in chondrocytes injury in OA. Similarly, our study also found that miR-495 played a malignant role in IL-1β-challenged chondrocytes by abating the protective role of circSERPINE2, which also indicated that circSERPINE2 could regulate chondrocytes injury in OA by modulating miR-495.

Next, we further explored the target of miR-495 in chondrocytes. Here we identified miR-495 could target TGFBR2, a gene with important role in chondrocyte phenotype [15]. In this research, we found that TGFBR2 expression was lower in OA patients and IL-1β-challenged chondrocytes, which was also like that in previous study [18]. Shen et al. suggested that TGFBR2 deletion could promote OA-like injury in mice [16]. Chen et al. reported that TGFBR2 was targeted via lncRNA MEG3 to suppress extracellular matrix degradation of chondrocytes [17]. Furthermore, Lu et al. TGFBR2 could suppress chondrocytes apoptosis in OA [18]. These previous reports have confirmed that TGFBR2 could attenuate chondrocytes apoptosis and extracellular matrix degradation induced via IL-1β. Hence, our study wanted to explore whether circSERPINE2 could target TGFBR2 to participate in regulating chondrocytes injury. Based on the crosstalk of miR-495 to circSERPINE2 and TGFBR2, we further identified that circSERPINE2 could promote TGFBR2 expression in chondrocytes via competitively binding with miR-495. Thus, we concluded that circSERPINE2 might target TGFBR2 by miR-495 to regulate chondrocyte injury.

In conclusion, circSERPINE2 alleviated IL-1β-induced chondrocytes apoptosis and extracellular matrix degradation, possibly via miR-495/TGFBR2 axis. This indicated a new mechanism for understanding the pathology of chondrocyte injury in OA, and provided circSERPINE2 as a target for OA treatment.

## Data Availability

The data and material presented in this manuscript are available from the corresponding author on reasonable request.

## Abbreviations

Ago2: anti-protein argonaute-2
AKT1: protein kinase Bα
circRNA: circular RNA
circSERPINE2: circRNA serpin family E member 2
COL2A1: collagen type II α 1
IL-1β: interleukin-1β
miRNA: microRNA
miR-495: microRNA-495
MTT: 3-(4,5-dimethyl-2-thiazolyl)-2,5-diphenyl-2–H -tetrazolium bromide
OA: osteoarthritis
PI: propidium iodide
qRT-PCR: quantitative reverse-transcription polymerase chain reaction
RIP: RNA immunoprecipitation
SOX9: Sry-type high-mobility-group box 9
TGFBR2: transforming growth factor-β receptor 2
